# Illinois State Dental Society

**Published:** 1867-10

**Authors:** 


### Proceedings of Societies.

---

THE semi-annual meeting of the Illinois State Dental Society, commenced its session in Chicago, May 14th, 1867, at 10 o'clock in the forenoon.

In the absence of Dr. H. N. Lewis, the President, Dr. O. Wilson, Vice-President, called the meeting to order, Dr. Smith, of Princeton, acting as Secretary.

After the usual preliminary business the following were elected members of the Society: Drs. A. W. French, Springfield; H. J. Smith, Quincy; J. Deschaur, Chicago; A. H. Day, Pekin; J. S. Marsh, Chicago; W. H. Truesdell, Elgin; Robert Gibson, Peoria; Alfred Shipley, St. Charles; W. C. Dyer, Chas. R. E. Koch, and E. D. Swain, Chicago.

The officers elected for the ensuing year were as follows:

*President*—Dr. G. H. Cushing, Chicago.

*Vice-President*—Dr. A. W. French, Springfield.

*Secretary*—Dr. M. S. Dean, Chicago.

*Treasurer*—Dr. J. N. Crouse, Mt. Carroll.

*Librarian*—Dr. W. W. Allport, Chicago.

*Executive Committee*—Drs. A. W. French, and S. Babcock, Springfield; H. N. Lewis, Quincy; L. F. Abbott, Wilmington; and E. H. Kilbourne, Aurora.

After which the meeting adjourned till 2 o'clock, P. M.

### AFTERNOON SESSION.

The newly elected president, Dr. G. H. Cushing, was escorted to the chair, and before assuming the duties of that office, briefly expressed his thanks to the Society for the honor conferred by his election, and hoped his conduct as presiding officer of the society would merit the confidence which the society had expressed by their selection.

After the reading of the minutes of the previous meeting, reports of committees were called for, and received.

Dr. Honsinger suggested that the hours from 9 to 11 each morning during the session, be devoted to clinical operations. The suggestion was favorably received, embodied in a resolution and adopted. Drs. Honsinger, Crouse and Brown, were appointed by the Chair as a committee of arrangements for that purpose.

Dr. French moved the adoption of the amendment of the By-laws, (as proposed by Dr. Ellis at the last regular meeting of the Society,) which was, to strike out section 2 of article 7, making the meetings of the society annual, instead of semi-annual, as heretofore. The motion was carried.

Some very interesting specimens of morbid anatomy were presented for the inspection of the society, by Drs. Abbott and Wilson.

The subject of *anæsthesia* was then announced by the Chair as the first subject in order for discussion, and was opened by Dr. Wilson, of Aurora, who read a concise and well written essay upon that subject, of which the following is an imperfect synopsis.

Anæsthesia by congelation possesses one strong point, namely, that in any event, in our profession, a fatal effect is highly improbable. "Dr. Richardson's is far in advance of Dr. Branch's method, but both fail to be universally efficient even in the majority of Dentists' hands, not because of the failure in the article used, but in its application. Chloroform is universally effective, ether nearly so. First effect of both is stimulating; the latter more than the former; this is of short duration. Then comes the effect sedative. The patient approaches death just in the ratio of the quantity absorbed at any one time; and the skill of the operator may be shown in *stopping* at the cessation of voluntary motion.

The more dense the nitrous oxyd gas, the more rapid the effect. The sense of something that is sometimes experienced by the patient, may be relieved by an occasional *natural* inhalation; ordinarily during the exhibition of gas the pulse will be accelerated one-third, "one hundred and twenty to the minute, full and hard, is generally considered a fatal pulse." "Up to this point it is not unusual to find it during the inhalation of protoxide of nitrogen, but what it makes up in rapidity it has lost in power." "If the pulse were full and increasingly rapid, this condition would burst the tubular bound, and effusion and apoplexy would ensue."

Dr. Kennicott discussed the subject fully, reviewing the different anæsthetic agents now in use, and gave some interesting facts in the early history of ether and chloroform, and his experiments in the manufacture and use of the latter as early as 1847. He prefers pure chloroform to any other anæsthetic now known. Formerly used ether and chloroform combined; but now re-distills and uses the latter exclusively, and considers it in the hands of scientific persons perfectly safe. Likes Richardson's spray producer for some cases; for front teeth and roots it answers a good purpose, and serves an admirable purpose for obtunding sensitive dentine. Thinks nitrous oxyd gas not only dangerous, but its introduction into the profession damaging in its effects.

Dr. Haskell did not use nitrous oxyd gas in practice, but has seen a good deal of it, and considers it the safest and best anæsthetic that has yet been discovered; has heard of no well authenticated case where it has been injurious in its results.

Dr. Gibson is willing to give nitrous oxyd gas a fair trial; thinks the *spray producer* beneficial in extracting dead teeth and old roots, but for teeth not deprived of their vitality, it produces more pain than it would to extract without it. Agrees with Dr. Kennicott, that "chloroform is a perfect God-send."

Dr. Kennicott said, cupidity and nitrous oxyd gas yoked together, were the most damnable things that were ever fastened to the human race; as valuable teeth were now extracted which might be saved by Dentists of the most ordinary capacity.

Dr. Day, of Pekin, had used the *spray producer*, but it had proved unsatisfactory. Is very much in favor of nitrous oxyd gas, and uses it extensively in his practice.

Dr. Smith, of Princeton, said that his patients informed him that the act of producing local anæsthesia by the *spray producer*, was more painful than the extracting of the tooth without it. Thinks it may serve a useful purpose in attracting the attention of the patient, but as an anæsthetic it amounts to nothing.

Dr. French, of Springfield, said, Branch's instrument for superficial roots worked admirably; but for casual extracting thought it nearly or quite useless; will not answer when the nerve is exposed; now used it occasionally for extracting the front teeth, but not for those posteriorly situated. Has had no trouble from sloughing. Does not use the gas (though he knows nothing against it), but relies principally on manual dexterity.

Dr. Wilson, of Aurora, thinks nitrous oxyd gas the safest and most useful of the anæsthetic agents. Because it is used by unskilled and dishonest men, as it often may be, is no reason why it should be discarded by the profession. If it is ever dangerous, it may be so in the advanced stages of decomposition of the lungs. Has heard unfavorable reports concerning its ultimate effects, but when traced to their source they are suddenly dissipated.

Dr. Kennicott, the chairman of the committee of arrangements reported that the proprietors of public amusements and art galleries, had kindly offered free tickets of admission to the Society during its session.

Adjourned until 11 o'clock to-morrow.

### SECOND DAY—AFTERNOON SESSION.

Owing to the unusual interest manifested in the clinical operations, the meeting adjourned until 2 o'clock, P. M.

Operations were performed by Dr. Smith, of Princeton; Forbes, of St. Louis; French, of Springfield, and Kennicott, of Chicago.

At 2 o'clock the meeting met pursuant to adjournment. Dr. Cushing in the chair.

After the usual preliminary proceedings, Drs. Judd, Eames, Forbes and Park, of St. Louis, Mo., who were present, were elected honorary members of the Society, and invited to take part in the discussions.

The subject of anæsthesia being passed, the president announced "*Filling Teeth*" the next in order.

Dr. Crouse said, this was the most important and the most difficult branch of our profession; considered gold the best material for fillings, and Woods' metal took rank next. In this operation moisture was the most difficult obstacle to overcome. The rubber dam and the wedge were important auxiliaries in this operation, but with these and the napkin it was often impossible to keep the materials perfectly dry until the operation was completed. Thought soft foil in the hands of ordinary operators generally the best; but for those more experienced, annealed foil might do better.

Dr. Forbes, of St. Louis, thought the profession might learn a great deal by inquiring into the causes of their failures. It was more humiliating, perhaps, to speak of these than of our successes; yet, as we all met with them, there was no disgrace in acknowledging it. In looking at the past, he was convinced that a majority of his failures was owing to the soft and friable parts of the teeth not being sufficiently and thoroughly cut away, or because the gold did not overlap the edges of the cavity. The first operation upon a decayed tooth was to probe it, in order to ascertain whether the nerve be exposed; then, to pass the excavator rapidly around the borders of the cavity where the enamel and dentine meet, taking great care that no soft spots or fissures are left to undermine the filling. To be perfectly assured of this the tooth should be thoroughly cleaned, so that the operator might more certainly detect them should they exist. He now uses the mallet and properly shaped chisels for the purpose of cutting away those portions which are necessary to be removed, as it is easier for the operator and more agreeable to the patient. Will not attempt to fill a cavity which he cannot examine in every part with the eye, as it is highly important that the operator should be able to see his work, in order that it may be done thoroughly.

Dr. Kennicott agrees in the main with Dr. Forbes, but thinks the delicate manipulator can readily determine by the sense of touch whether the decay is thoroughly removed, and that the nature of the case sometimes rendered it impossible to examine every portion of the cavity by any other means.

Dr. French, of Springfield, thinks he was formerly too careful about cutting the tooth away sufficiently, that the operator must have room enough to examine the cavity, and to introduce the gold properly. He used very small napkins placing several of them in the mouth at a time, and when the secretions rendered it necessary removed the lower one and placed a dry one over those remaining. Should the gold become moistened before the operation is complete, he places a dry napkin over the filling and drives it out by the plugger and mallet.

Dr. Honsinger attributes many of his failures, especially in filling the bicuspids, to a desire to preserve *all* of the tooth. Such teeth had become broken down with use, and in this way he formerly lost many. He now cuts away more freely these portions which are weak and friable, and for this purpose uses the chisel more and the file less. Protests against the practice of forcibly wedging, as it sometimes destroys and breaks up the tissues that intervene. Had witnessed several cases of this kind; generally used rubber for separating the teeth, which should be changed every few days.

Dr. Eames, of St. Louis, thinks the important point is to keep the work perfectly dry, detailed his method of using the rubber dam, by securing it with a waxed linen or silk thread. Also demonstrated the manner of using a very ingenious instrument gotten up by himself, the *Tongue holder and duct compresser.* This instrument was very favorably regarded by the society, and was considered a valuable improvement. Prepared his gold in ribbons, using No. 4, Dunlevy's foil, and in square blocks; in size a little more than the depth of the cavity. These he first secured in the cavity, allowing them to project, or overlap the margins, and lastly uses the annealed ribbons to finish up the work.

The subject of *Filling Teeth* was now dismissed, and that of "*The Treatment of Exposed Nerves*" taken up.

Dr. Crouse said the first thing to be done when the nerve was found exposed was to destroy with creosote and arsenic and remove it. Thought the incautious manner of using arsenic, allowing it to come in contact with the soft parts, by not securely cementing it within the cavity, the cause of soreness in the teeth which frequently followed its use. To secure it in the cavity he used cotton and sandarac varnish, and leaves it in twenty-four hours, though it can do no injury when well secured, if left months.

Dr. Gibson said, arsenic should not be allowed to remain in the tooth for a longer time than it takes to accomplish the object desired. Thinks it is injurious to the tooth if allowed to remain too long; that after it has performed its duty in destroying the nerve pulp, it commences on the tooth itself; thought we often doctored too much.

Dr. Ellis said arsenic should be used cautiously, but in small quantities it was not dangerous. Finds nerve pulps which resist its action for a long time, especially if they possess a low vitality or are bleeding. Saw a severe case of poisoning from its careless use, in California, in which the alveolar processes were so much affected, that they became necrosed in consequence, and the patient lost not only the tooth to which it was applied, but the adjoining ones also. He uses as little as possible.

Dr. Kennicott uses a sedative composed of sulphate of morphia and aconitine dissolved in creosote, which he uses in combination with an aqueous solution of arsenic. Places cotton over them and seals tightly with softened wax.

Dr. Judd, of St. Louis, thinks in some instances arsenic will permeate the dentine; much more in some teeth than in others. For example, it will destroy the nerve pulp which is not exposed, when it is placed in the cavity of a tooth. Has seen by the aid of the microscope, channels which extended through both the cementum and dentine. In such cases the arsenic might pass through these tissues, and cause inflammation of the peri-cementum. Thought this a more reasonable supposition than that it is absorbed by the blood vessels of the pulp, and conveyed through the foramen in the apex of the root. Thought Dr. Ellis might be mistaken in regard to arsenic being the cause of disease, in the case which he related, and that it was necrosis resulting from some other cause or causes.

Dr. Cushing thought that the necrosis might have resulted from alveolar abscess, instead of arsenic.

On motion of Dr. French, the rules were suspended in order to elect delegates to the *American Dental Association.*

Drs. Haskell, Kennicott and Smith, of Quincy, were appointed a nominating committee.

Adjourned until 11 o'clock the next day.

THIRD DAY—MORNING SESSION.

Society met at 11 A. M. After the usual preliminaries, the President announced *Alveolar Abscess* as the next topic for discussion.

Dr. Ellis thought it unwise to undertake to cure promiscuous cases of alveolar abscess. He would select such patients as would appreciate a successful operation, and who were willing to pay for it, and who in case of failure would not be unreasonable in their condemnation. The people must be taught that ours, like the medical profession, is not an infallible one, and that our work may fail. Treats more generally with creosote and iodine, and in these, as in other operations is careful to say it *may fail.* The warranting of operations he considers highly unprofessional and never does it. Thinks there is danger of filling too soon after treatment, and often treats after he considers the abscess cured. Fills the nerve cavity invariably with cotton saturated with creosote.

Dr. Freeman thinks the too free use of creosote and odine, where there is not a fistulous opening, apt to create too much inflammation.

Dr. Eames sometimes drills through the process in order to break up the sac, but would not resort to this until other means had been tried.

Dr. Judd, in the first stages of the disease attempts to reduce the inflammation by the aid of leeches or warm applications. After pus is formed it *may* be absorbed, *but never as pus.* After an opening in the process has been made, it may be filled up with cicatrized tissue. When pus makes its exit along the side of the tooth, it is much more difficult to cure, than when it passes through the nerve cavity. To destroy the sac, when formed, force creosote or iodine through the cavity and the inflammatory action will destroy it. There are other complications more difficult to treat. When the cementum of the root is absorbed, leaving the exposed dentine as a source of irritation. In such cases the apex of the root should be excised.

Dr. Nichols was pleased with Dr. Chase's remedy, *mercurius vivus,* and advised its trial.

Dr. Reeber thought Dr. Chase claimed too much for his remedy; but that it was, nevertheless, worthy of a trial. He had used it with some success.

Dr. Kennicott related an interesting case of necrosis of the lower jaw. Removed a large portion of the necrosed bone about one inch in length, and although a great portion of the periosteum was destroyed, new and healthy bone had been formed.

Dr. Honsinger related a severe case of necrosis of the upper jaw. Removed the larger portion of the bone between the eye teeth, yet ulceration continued, and on examination found the former so much diseased, that he had to remove it. He finally discovered that it extended to the antrum, which after extracting the bicuspids he laid bare by removing the necrosed bone. The patient he afterwards learned, was receiving at the same time, constitutional treatment for syphilis.

Society adjourned until 2 o'clock, P. M.

AFTERNOON SESSION.

The subject of *Alveolar Abscess* was passed, and *Dental Neuralgia* taken up.

Dr. Judd remarked that he knew little of the pathology of that disease, as the subject was involved in great mystery. This, of all other diseases, by medical practitioners had been treated the most empirically. The causes were generally local irritation, and a vast majority of cases were more or less, directly or remotely connected with the teeth. Thinks the medical practitioner who treats neuralgia without examining the condition of the mouth, understands his business but poorly.

Dr. Park, of St. Louis, had used nitrous oxyd gas in a few cases which had given instant, and as far as he knows permanent relief.

Dr. Gibson thought local irritation the general cause of that disease, and that the treatment required the removal of that cause. Exostosis of the roots, as well as disorganized nerves was often the cause of neuralgia.

Dr. Kennicott agrees that exostosis is the prevailing cause of neuralgia, though the causes were often remote. Related a case now under treatment, which was caused by uterine irritation. His remedies were *anti-periodics*. Cited an interesting case of long standing which he had cured by severing the supra orbital nerve. The patient had received a blow in that region, over twelve years before, which had undoubtedly fractured the bones causing them to press upon that nerve.

Dr. Forbes, of St. Louis, in 1837 extracted several sound teeth for a lady, by the *order* of a physician, without affording relief. The patient, to this day, can find relief only in *blue-mass.*

Dr. French often finds the wisdom teeth the offenders, and that the others suffer from sympathy; by removing these the pressure was relieved, and a cure effected.

Dr. Kennicott stated the case of a man whose teeth were much crowded, and who suffered excruciating neuralgic pains. A cure was instantly effected by passing a thin separating file between the first molar and bicuspid.

Dr. Baker also related an interesting case in practice.

On motion of Dr. Crouse, the subject of Dental neuralgia was dismissed and that of *sensitive dentine* introduced.

Dr. Freeman has used cautiously creosote and arsenic as a remedy, allowing it to remain from 5 to 24 hours, without witnessing any bad results.

Dr. Crouse believes a sharp excavator the best remedy for sensitive dentine. Prepared chalk placed around the gums at night often affords relief. Sometimes fills with Hill's stopping allowing it to remain a few weeks.

Dr. Gibson thinks the excavator perhaps the best remedy. Has tried most of the remedies used for that purpose excepting aconite, but has had the best success with arsenic. Thinks when the patient is under the control of the Dentist, that there is little danger to be apprehended from its careful use.

Dr. Cushing rather unwillingly used arsenic in one case. The cavity was superficial and the quantity used, small. About a year afterwards inflammation ensued, and upon opening to the nerve pulp found it much congested.

Dr. Eames had used arsenic with unpleasant results. Should not be used in teeth of children. In excavating he cuts *from* the nerve; uses glycerole of tannin.

Dr. Judd thought, in cases where the absorption was feeble and the teeth dense, there might be little danger in using arsenic.

Dr. Honsinger used it until he formed an unfavorable opinion of it. Thought it too dangerous a remedy to be generally used.

Dr. Clarke, of Beloit, Wis., said, to cut outward with a sharp instrument succeeds about as well as anything else. The best way is to cut *through* the decay at once; when extremely sensitive fills with cotton. This with an alkaline treatment for a few weeks will remove the sensitiveness entirely.

Dr. Haskell, chairman of the committee to nominate delegates to the American Dental Association, reported the following nominees:

Drs. E. H. Kilbourne, Aurora; A. W. French, Springfield; W. W. Allport, Chicago; Robert Gibson, Peoria; J. A. Kennicott, Chicago; J. A. Crouse, Mt. Carroll; J. P. Foltz, Mendota; H. J. Smith, Quincy; A. H. Day, Pekin; A. S. Reeber, Chicago; L. F. Abbott, Wilmington; and W. Albaugh, Chicago, which were duly elected.

Dr. Cushing made the following statement in regard to the proceedings of the Goodyear Vulcanite Company. The recent decision of the Courts in the various cities where applications for injunctions have been made against Dentists, to restrain them from using the vulcanized rubber in their practice, have been made use of by the Goodyear Dental Vulcanite Company, to intimidate the profession into the acknowledgment and settlement of their claims. Many Dentists have the impression that they can be compelled to settle these claims without further steps being taken against them. This impression should be removed. No Dentist is called upon to settle any such claim until he may be sued, and any Dentist so sued will only be called upon by the Courts to give bonds to pay the royalty, provided the claims of the company are sustained by the Supreme Court; and any person who belongs to, or may become a member of the association for defending suits, will be provided with counsel by the association.

Dr. Kennicott, chairman of committee of arrangements, in behalf of the officers of the Academy of Design, tendered an invitation to the members of the society to a collation at their rooms this evening.

On motion, the President appointed Drs. French, of Springfield; and Smith, of Quincy, as a committee to secure essayists, who should prepare a brief paper to be read in opening each topic for discussion at the next meeting.

FOURTH DAY—MORNING SESSION.

The meeting was called to order by the President, Dr. Cushing, at 9 o'clock in the forenoon.

Dr. R. Gibson, of Peoria, was appointed to deliver the address at the next meeting of the society.

Dr. Haskell read an interesting paper on *continuous gum work*, setting forth its superior advantages over other styles of work, and urging the importance of competent instruction in this branch of mechanical Dentistry at our Dental Colleges.

The subject of *impressions* and casts was taken up and discussed by Drs. Wilson, Kennicott, Haskell, Kilbourne, Smith, Reeber, and others.

Dr. Kennicott offered the following resolution which was carried.

*Resolved*, That this Society unqualifiedly condemns the practice by members of the Dental profession, of taking students to be sent forth to practice upon a confiding community, after only from three months to one year's study and for a pecuniary consideration, and would recommend that students be taken for a term of not less than three years of study, in addition to graduation.

The society adjourned at 12.30 P. M., at which time the interest of the meeting continued unabated. Its next session will be held at Springfield, on the second Tuesday of May next.

